# Preconceptional Lipid-Based Nutrient Supplementation in 2 Low-Resource Countries Results in Distinctly Different IGF-1/mTOR Placental Responses

**DOI:** 10.1093/jn/nxaa354

**Published:** 2020-12-31

**Authors:** Marisol Castillo-Castrejon, Ivana V Yang, Elizabeth J Davidson, Sarah J Borengasser, Purevsuren Jambal, Jamie Westcott, Jennifer F Kemp, Ana Garces, Sumera A Ali, Sarah Saleem, Robert L Goldenberg, Lester Figueroa, K Michael Hambidge, Nancy F Krebs, Theresa L Powell

**Affiliations:** Division of Reproductive Sciences, Department of Obstetrics and Gynecology, University of Colorado Anschutz Medical Campus, Aurora, CO, USA; Biomedical Informatics & Personalized Medicine, Department of Medicine, University of Colorado Anschutz Medical Campus, Aurora, CO, USA; Biomedical Informatics & Personalized Medicine, Department of Medicine, University of Colorado Anschutz Medical Campus, Aurora, CO, USA; Section of Nutrition, Department of Pediatrics, University of Colorado Anschutz Medical Campus, Aurora, CO, USA; Section of Nutrition, Department of Pediatrics, University of Colorado Anschutz Medical Campus, Aurora, CO, USA; Section of Nutrition, Department of Pediatrics, University of Colorado Anschutz Medical Campus, Aurora, CO, USA; Section of Nutrition, Department of Pediatrics, University of Colorado Anschutz Medical Campus, Aurora, CO, USA; Maternal and Infant Health Center, Institute of Nutrition of Central America and Panama (INCAP), Guatemala City, Guatemala; Department of Community Health Sciences, Aga Khan University, Karachi, Pakistan; Department of Community Health Sciences, Aga Khan University, Karachi, Pakistan; Department of Obstetrics and Gynecology, Columbia University, New York, NY, USA; Maternal and Infant Health Center, Institute of Nutrition of Central America and Panama (INCAP), Guatemala City, Guatemala; Section of Nutrition, Department of Pediatrics, University of Colorado Anschutz Medical Campus, Aurora, CO, USA; Section of Nutrition, Department of Pediatrics, University of Colorado Anschutz Medical Campus, Aurora, CO, USA; Section of Neonatology, Department of Pediatrics, University of Colorado Anschutz Medical Campus, Aurora, CO, USA

**Keywords:** maternal–fetal exchange, nutrition, zinc, pregnancy, stunting

## Abstract

**Background:**

Preconceptional maternal small-quantity lipid-based nutrient supplementation (SQLNS) improved intrauterine linear growth in low-resource countries as demonstrated by the Women First Preconception Maternal Nutrition Trial (WF). Fetal growth is dependent on nutrient availability and regulated by insulin-like growth factor 1 (IGF-1) through changes in placental transfer capacity, mediated by the mechanistic target of rapamycin (mTOR) pathway.

**Objectives:**

Our objective was to evaluate the role of placental mTOR and IGF-1 signaling on fetal growth in women from 2 low-resource countries with high rates of stunting after they received preconceptional SQLNS.

**Methods:**

We studied 48 women from preconception through delivery who were from Guatemala and Pakistan and received SQLNS or not, as part of the WF study. Placental samples were obtained at delivery (control, *n* = 24; SQLNS, *n* = 24). Placental protein or mRNA expression of eukaryotic translation initiation factor binding protein-1 (4E-BP1), ribosomal protein S6 (rpS6), AMP-activated protein kinase α (AMPKA), IGF-1, insulin-like growth factor receptor (IGF-1R), and pregnancy associated plasma protein (PAPP)-A, and DNA methylation of the *IGF1* promoter were determined. Maternal serum IGF-1, insulin-like growth factor binding protein (IGFBP)-3, IGFBP-4, IGFBP-5, PAPP-A, PAPP-A2, and zinc were measured.

**Results:**

Mean ± SEM maternal prepregnancy BMI differed between participants in Guatemala (26.5 ± 1.3) and Pakistan (19.8 ± 0.7) (*P* < 0.001). In Pakistani participants, SQLNS increased the placental rpS6(T37/46):rpS6 ratio (1.5-fold) and decreased the AMPKA(T172):AMPKA ratio. Placental *IGF1* mRNA expression was positively correlated with birth length and birth weight *z*-scores. Placental PAPP-A (30-fold) and maternal serum zinc (1.2-fold) increased with SQLNS. In Guatemalan participants SQLNS did not influence placental mTOR signaling. Placental IGF-1R protein expression was positively associated with birth length and birth weight *z*-scores. SQLNS increased placental PAPP-A (40-fold) and maternal serum IGFBP-4 (1.6-fold).

**Conclusions:**

In Pakistani pregnant women with poor nutritional status, preconceptional SQLNS activated placental mTOR and IGF-1 signaling and was associated with improved fetal growth. In contrast, in Guatemalan women SQLNS did not activate placental nutrient-sensing pathways. In populations experiencing childhood stunting, preconceptional SQLNS improves placental function and fetal growth only in the context of poor maternal nutrition. This trial was registered at clinicaltrials.gov as NCT01883193.

See corresponding commentary on page 471.

## Introduction

Linear growth is an indicator of a child's well-being and is an accurate marker of population inequalities in human development (1). Low birth weight (LBW) is common in undernourished populations in low- and middle-income countries (LMICs) and has been associated with childhood stunting. Stunting has been identified as a major global public health problem with short- and long-term health consequences, such as deficits in cognitive function and decreased adult economic productivity (2). Overall, it has been estimated that 20% of childhood stunting could have its origins in the fetal period (3). The pathogenesis underlying linear growth failure is not well established, but it likely begins in utero and continues into postnatal life (4, 5).

Short maternal stature, suboptimal maternal nutrition, and poor weight gain during pregnancy are major factors associated with LBW (2, 6). Human and animal studies have suggested a beneficial effect on birth outcomes when maternal underweight and micronutrient deficiencies are improved before conception (7, 8). Recently, the Women First Preconception Maternal Nutrition Trial (WF) demonstrated that poor fetal growth in low-resource countries, including linear growth, can be improved with maternal nutritional supplementation initiated before conception or late in the first trimester (9).

Nutrient transfer across the placenta is critically important for fetal growth and programs the fetus for diseases later in life (10). The placenta is known to adapt its structure and function in response to maternal fitness, thereby affecting fetal growth (11, 12). We have previously demonstrated that the placenta acts as a nutrient sensor, highlighting the role of mechanistic target of rapamycin (mTOR), which regulates energy-requiring processes including growth, nutrient transport, and protein synthesis (13).

In humans, maternal concentration of insulin-like growth factor-1 (IGF-1) is positively correlated with birth weight (14). IGF-1, insulin-like growth factor receptor (IGF-1R), and IGF binding proteins (IGFBPs) are expressed in the placenta. In vivo and in vitro studies have shown endocrine and autocrine/paracrine actions of IGF-1 in regulating fetal growth and placental transport capacity (15, 16).

IGF-1 activity is regulated by pregnancy-associated plasma protein (PAPP)-A and PAPP-A2, zinc binding metalloproteinases (17). Lack of PAPP-A activity increases IGFBP-4 concentrations, resulting in an ∼40% decrease in fetal growth in mice (18, 19) and intrauterine growth restriction in humans (20). Prenatal maternal zinc supplementation is associated with greater fetal weight in a low-resource population (21) and zinc supplementation in zinc-deficient short children increased plasma IGF-1 (22, 23).

Alterations in DNA methylation in the promoter of the *IGF1* gene have been associated with fetal growth. Placentas from growth-restricted newborns have higher levels of DNA methylation in the promoter regions of the *IGF1* gene than those from newborns with normal growth (24). High levels of DNA methylation of the P2 promoter of the *IGF1* gene in mononuclear blood cells were observed in children with idiopathic short stature (25) and negatively correlated with serum IGF-1 concentration and child height (26).

Based on the divergent impact of preconceptional small-quantity lipid-based nutrient supplementation (SQLNS) on birth length in Guatemala and Pakistan in the WF trial, we sought to determine the effect of maternal preconceptional SQLNS on placental mTOR and IGF-1 signaling and their association with fetal growth in these 2 low-resource countries where stunting is prevalent.

## Methods

### Study design

This study was part of a randomized controlled trial registered as Women First (NCT01883193), which was designed to determine the optimal timing of maternal nutrition supplementation to improve fetal linear growth. The trial was conducted in rural and semirural locations in Guatemala (Chimaltenango Department) and Pakistan (Thatta, Sindh Province) where stunting rates are high (27).

### Participants

The present study focused on a subcohort of Guatemalan and Pakistani women from whom ∼50 placentas were collected at each site. Women signed informed consent for participation, which was approved by the Colorado Multiple Institutional Review Board, University of Colorado, and by the local and/or national ethics committees. Women were randomly assigned to a daily small-quantity lipid-based micronutrient supplementation (SQLNS, Nutriset) starting ≥3 mo before conception and continuing until delivery (preconceptional SQLNS, arm 1) or no nutritional supplementation at all (control group, arm 3). SQLNS is a commercially available supplement developed for low-resource settings that has been used to enrich the diets of pregnant women (28, 29). The composition has been described in detail in the published WF protocol (27). Women taking SQLNS were also provided with an additional daily lipid-based protein-energy supplement (no additional micronutrients) under 2 conditions: BMI (in kg/m^2^) <20 at any time while receiving SQLNS or inadequate gestational weight gain based on the Institute of Medicine guidelines (30). This second supplement provided 300 kcal and 12 g protein; recipients were encouraged to consume ≥50% without leading to reduced intake of their habitual diet. Women in the control group (arm 3) received no nutritional supplements. Compliance was documented by biweekly collection of empty sachets, self-reported consumption, and random audits by on-site personnel. Compliance for this study was calculated by the total number of sachets fully eaten divided by the total number of days between starting supplementation and delivery, reported as a percentage.

Participants whose placenta was collected at delivery from each site (*n* = 50, convenience sample) were categorized into quartiles according to their newborns’ birth length. A total of 12 participants from the first and fourth quartiles per treatment arm and site (*n* = 24/site) were included in the study. Gestational age was confirmed by first-trimester ultrasound crown–rump length and only pregnancies with term deliveries (>37 weeks of gestation) were included. Newborn length and weight were obtained within 48 h of birth by trained study personnel. Gestational-age-adjusted length and weight were determined based on INTERGROWTH-21st fetal growth charts (31).

### Tissue collection

Placental dimensions were obtained after delivery by alignment of the longest axis of the placenta (width) on a grid and recording the widest point perpendicular to the longest dimension axis (length). Placental tissue was collected from 4 representative locations (1 cm^3^ each), washed, and immersed into PBS with protease inhibitors or RNAlater^TM^ (Thermo Fisher Scientific). Samples were stored at −80°C until further analysis. Collection time and storage temperature were recorded until samples were shipped to the University of Colorado, Denver, USA.

### Placental protein expression

Placentas were thawed and homogenized in PBS solution containing protease inhibitor (1:100; Sigma Aldrich Cat. No. P8340) and phosphatase inhibitor cocktails 2 and 3 (1:100; Sigma Aldrich Cat. Nos. P5726, P0044). Equal amounts of total protein (1 μg) from placental homogenates were used to determine the total and phosphorylated proteins using the automated capillary electrophoresis Simple Western system (Protein Simple, Cat. Nos. SM-W004-1, PS-ST01, PN-009-050, DM-001). Proteins were separated and probed using primary antibodies for total AMP-activated protein kinase α (AMPKA), AMPKA(T172), total ribosomal protein S6 (rpS6), rpS6(S235/236), total eukaryotic translation initiation factor binding protein-1 (4E-BP1), 4E-BP1(T37/46), IGF-1R, PAPP-A, and zinc influx SLC39A transporter (SLC39A10). Vinculin or β-actin served as the loading controls (**Supplemental Table 1**). Analysis was performed by Compass (Protein Simple, San Jose, CA, 95134) software (32). For comparison purposes the mean protein expression of placentas from nonsupplemented women (control) was assigned a value of 1.0 and used as a reference.

### Placental *IGF1* mRNA expression

Total placental RNA was extracted using an RNase Mini Plus kit (Qiagen, Cat. No. 74136) after homogenization of 100 mg placental tissue in 900 µL TRI Reagent (Life Technologies, Cat. No. 15596-026). Quantity and quality of total RNA were analyzed using a NanoDrop 2000/2000c spectrophotometer (Thermo Fisher Scientific). Reverse transcription was performed using 1.0 µg total RNA according to the manufacturer's protocol (High-Capacity cDNA Reverse Transcription Kit with RNase Inhibitor, Applied Biosystems, Cat. No. 4374966).

Expression of the *IGF1* gene (Hs01547656_m1) was determined in placental samples by qRT-PCR using Taqman probes (TaqMan Gene Expression Assay-Standard Condition, Applied Biosystems, Cat. No. 4370048) in duplicate and analyzed using the QuantStudio 6 Flex system (Applied Biosystems). Relative expression of the *IGF1* gene was normalized to the geometric mean of 18S ribosomal RNA (RNA18S5, Hs03928985_g1) and 3-monooxygenase/tryptophan 5-monooxygenase activation protein ζ (YWHAZ, Hs01122445_g1) using the ΔΔCT method. Endogenous reference gene expression was similar in placentas from control and supplemented women.

### Placental DNA methylation

The P2 promoter region of the *IGF1* gene was assessed based on a previous association between higher DNA methylation in this regulatory element and human early growth (26). We focused on an area of open chromatin in the P2 promoter, as defined by the presence of H3K27 acetylation and DNase hypersensitivity (**Supplemental Figure 1**). The DNA sequence from this region was analyzed using PyroMark Assay Design Software (Qiagen) and the 2 highest-performing primer sets were selected. Bisulfite-converted DNA was PCR amplified with 1 biotinylated PCR primer. A sequencing primer was then added, which anneals to the single-stranded DNA template. Pyrosequencing was performed on the PyroMark Q96 MD sequencer (Qiagen) using reagents and protocols recommended by the manufacturer. Each plate contained 0%, 50%, and 100% methylated controls. Percentage methylation was calculated from the peak heights of C and T using the Pyro Q-CpG software (Qiagen). PCR and sequencing were performed in duplicate for each sample and mean values were used in the statistical analysis.

### Measurements of maternal serum hormones and zinc concentrations

Maternal serum concentrations of total IGF-1, IGFBP-3, IGFBP-4, IGFBP-5, PAPP-A, and PAPP-A2 at 34 weeks of gestation were determined by a solid-phase quantitative sandwich enzyme immunoassay technique (R&D Systems, Cat. Nos. DG100, DGB300; Ash Labs, Cat. Nos. AL-128, AL-127, Al-106, AL-109). Samples were run in duplicate. A pooled serum sample and a commercial quality control sample (R&D Systems, Cat. No. QC22) were used to evaluate reproducibility. Concentrations (ng/mL or mIU/mL) were calculated using a known standard curve run on each plate.

Maternal serum zinc concentration at 34 weeks of gestation was determined by inductively coupled plasma MS using Agilent Technologies model 7700× (Agilent Technologies). Precautions were taken to avoid zinc contamination during the collection, handling, and storage of specimens. Concentrations (μg/dL) were calculated based on a commercial standard solution used to generate a standard curve (Sigma Aldrich TraceCERT, Cat. No. 75594).

### Data presentation and statistical analysis

Participants’ baseline characteristics were compared using 1-factor ANOVA and Tukey's multiple comparisons test. Comparisons between the maternal preconceptional SQLNS and control groups were evaluated by Student's unpaired *t* test. The data complied with homoscedastic or normality assumptions. In order to further explore underlying factors determining fetal growth trajectories in these 2 populations, control and preconceptional SQLNS data were pooled and Pearson's correlation unadjusted models were used to examine associations between placental protein expression, maternal serum proteins, and fetal growth parameters. A *P* value < 0.05 and α = 0.05 were considered statistically significant. Data are presented as mean ± SEM. Statistical analysis was performed using Prism 8.0 software (GraphPad Software, Inc.).

## Results

### Participant characteristics

Selected clinical characteristics are presented by site and stratified by treatment arm (**Table 1**). There were no statistical differences between women in the 2 treatment arms for maternal age, height, and weight gain at 34 weeks of gestation or parity. Participants from Guatemala started pregnancy with a significantly higher preconceptional BMI than Pakistani participants. Within each study site preconceptional BMIs between each treatment arm were not different.

**TABLE 1:** Baseline characteristics of study participants, presented by site and treatment arm^1^

	Guatemala study site		Pakistan study site		
	Control (arm 3)	Preconceptional SQLNS (arm 1)	Control (arm 3)	Preconceptional SQLNS (arm 1)	*P* value
Mother					
Maternal age, y	25.2 ± 1.50	25.0 ± 1.18	24.0 ± 1.20	24.4 ± 1.13	NS
Parity	1.66 ± 0.33	1.33 ± 0.25	1.58 ± 0.54	1.75 ± 0.47	NS
Nulliparity, *n*	1	2	5	3	NS
BMI, kg/m^2^	26.5 ± 1.31^a^	26.3 ± 1.33^a^	19.8 ± 0.68^b^	19.7 ± 0.70^b^	<0.001
Underweight*, n*	0	0	4	5	
Normal*, n*	6	5	7	6	
Overweight, *n*	4	4	1	1	
Obese, *n*	2	3	0	0	
Height, cm	147 ± 1.03	147 ± 1.36	152 ± 2.25	148 ± 2.03	NS
Weight, kg	57.5 ± 3.26^a^	57.0 ± 2.83^a^	46.2 ± 2.22^b^	43.9 ± 1.10^b^	<0.01
Gestational age at delivery, wk	38.9 ± 0.35	39.4 ± 0.27	39.0 ± 0.32	39.4 ± 0.45	NS
Weight gain at 34 wk, kg	5.41 ± 1.19	5.33 ± 1.38	6.33 ± 1.88	5.66 ± 1.53	NS
Mode of delivery, *n*					
Vaginal	9	7	12	12	
Cesarean	3	5	0	0	
SQLNS compliance, %	—	79.0 ± 4.50	—	91.1 ± 2.89	0.01
Protein supplement frequency, *n*	—	2/12	—	10/12	<0.0001
Newborn					
Birth weight, kg	2.83 ± 0.11	2.98 ± 0.11	2.81 ± 0.12	2.70 ± 0.16	NS
Birth length, cm	48.0 ± 0.59	48.0 ± 0.69	47.2 ± 0.92	48.0 ± 0.81	NS
Length-for-gestational-age *z*-score	−0.79 ± 0.33	−0.52 ± 0.39	−1.05 ± 0.51	−0.67 ± 0.50	NS
Weight-for-gestational-age *z*-score	−1.01 ± 0.24	−0.68 ± 0.29	−1.05 ± 0.25	−1.41 ± 0.37	NS
Sex, *n*					
Male	6	6	5	5	
Female	6	6	7	7	
Placental area, cm^2^	162 ± 10.3^a^	176 ± 9.70^a^	82.8 ± 14.7^b^	115 ± 5.56^c^	<0.05

1 Values are means ± SEMs, *n* = 12. Labeled means in a row without a common letter differ, *P* < 0.05. SQLNS, small-quantity lipid-based nutrient supplementation.

Guatemalan participants reported 70% compliance in consuming SQLNS (Supplement 1) from before conception until delivery, which was significantly lower than Pakistani participants, who reported 91% compliance. Supplement compliance was not identified as a factor in fetal growth outcomes (9). All Pakistani participants in this subcohort received Supplement 2 and reported high compliance (>90%). Only 2 Guatemalan participants received Supplement 2.

In this cohort of participants, there were no significant differences between sites and treatment arms in birth weight, birth length, length-for-gestational-age *z*-scores, or weight-for-gestational-age *z*-scores. Placental area was not statistically different between treatment arms in Guatemalan participants. Overall, Pakistani participants had significantly smaller placental areas than Guatemalan participants; however, Pakistani mothers who received preconceptional SQLNS had a significantly larger (+1.4-fold, *P* = 0.03) placental area than the control arm and placental area was correlated with length-for-age *z*-score (*P* = 0.05, *r* = 0.39).

### Effect of preconceptional SQLNS on the placental mTOR signaling pathway

**FIGURE 1:**
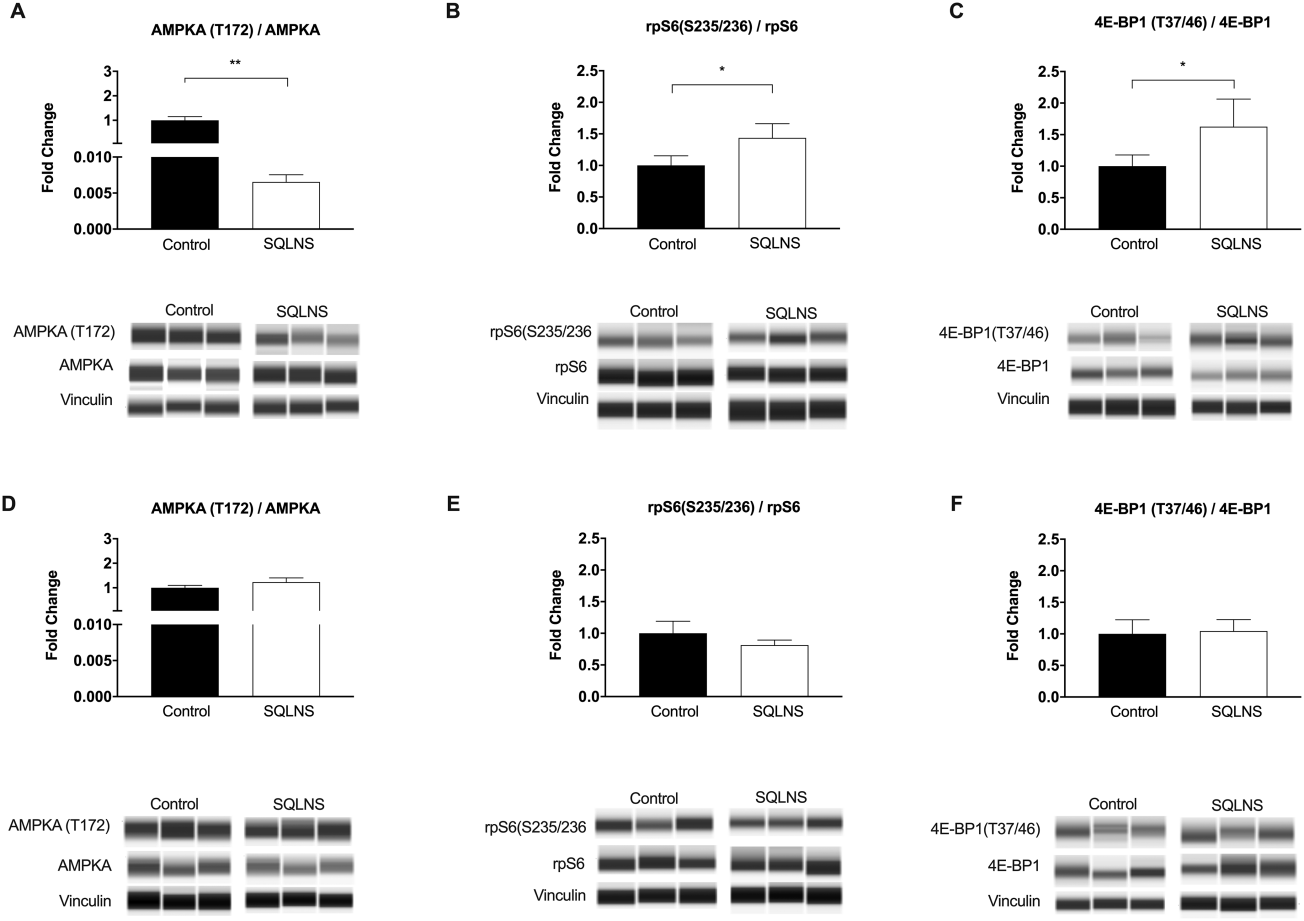
Effect of preconceptional SQLNS on placental AMPK and mechanistic target of rapamycin signaling in Pakistan (A–C) and Guatemala (D–F). The histogram shows the relative protein expression of the (A, D) AMPKA (T172):total AMPKA ratio, (B, E) rpS6 (S235/236):total rpS6 ratio, and (C, F) 4E-BP1 (T37/46):4E-BP1 ratio. Representative individual Protein Simple Western Blot capillaries are shown for total and phosphorylated proteins and vinculin as loading control. Data are presented as mean ± SEM, *n* = 12. ^*,**^Different from control: *P < 0.05, **P < 0.005 (Student's unpaired *t* test). AMPKA, AMP-activated protein kinase α; rpS6, ribosomal protein S6; SQLNS, small-quantity lipid-based nutrient supplementation; 4E-BP1, eukaryotic translation initiation factor binding protein-1.

In Pakistani participants, in terms of protein expression, preconceptional SQLNS decreased the placental AMPKA(T172):AMPKA ratio (**Figure 1**A) (*P* = 0.001) and increased the placental rpS6(S235/236):rpS6 ratio (Figure 1B) (+1.5-fold, *P* = 0.03) and 4E-BP1(T37/46):4E-BP1 ratio (Figure 1C) (+1.6-fold, *P* = 0.09) as compared with control participants. Collectively these results suggest an inhibition of AMPK signaling and an activation of mTOR signaling in women receiving SQLNS. In contrast, in placentas from Guatemalan participants preconceptional SQLNS had no effect on AMPK or mTOR signaling activity, as evidenced by no difference in the placental AMPKA(T172):AMPKA ratio (Figure 1D), rpS6(S235/236):rpS6 ratio (Figure 1E), or 4E-BP1(T37/46):4E-BP1 ratio (Figure 1F) between the 2 treatment arms. We found no significant correlations between placental mTOR signaling and length-for-age *z*-score in either study site [AMPKA(T172):AMPKA ratio: Guatemala *P* = 0.49, *r* = −0.002; Pakistan *P* = 0.46, *r* = −0.02; rpS6(S235/236):rpS6 ratio: Guatemala *P* = 0.22, *r* = 0.26; Pakistan *P* = 0.40, *r* = 0.06; 4E-BP1(T37/46):4E-BP1 ratio: Guatemala *P* = 0.97, *r* = 0.008; Pakistan *P* = 0.84, *r* = 0.04].

### Effect of preconceptional SQLNS on placental expression of *IGF1* and maternal serum concentration of IGF-1 and its association with fetal growth

**FIGURE 2:**
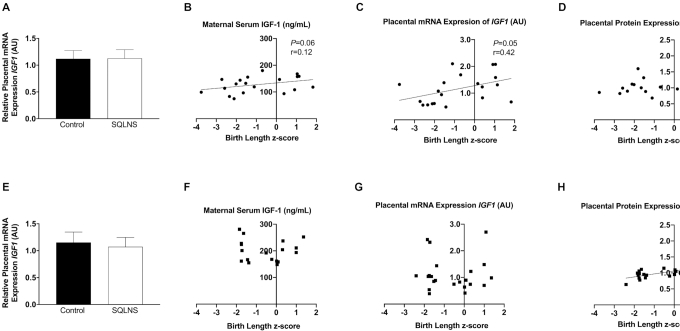
Effect of preconceptional SQLNS on, and the association of the IGF-1 axis with, birth length in Pakistani (A–D) and Guatemalan (E–H) study sites. (A, E) The histogram shows relative placental *IGF1* mRNA expression by treatment arm (control, arm 3; SQLNS, arm 1) and site. Data are presented as mean ± SEM, *n* = 12. Pearson's correlation of (B, *n* = 19; F, *n* = 20) maternal serum concentration of IGF-1, (C, *n* = 19; G, *n* = 22) relative placental *IGF1* mRNA expression, and (D, *n* = 19; H, *n* = 22) placental protein expression of IGF-1R with birth length *z*-score. AU, arbitrary units; IGF-1, insulin-like growth factor-1; IGF-1R, insulin-like growth factor receptor; SQLNS, small-quantity lipid-based nutrient supplementation.

Preconceptional SQLNS did not affect the relative mRNA expression of *IGF1* in placental tissues for either site (**Figure 2**A, E). In Pakistani participants, we found a borderline significant positive correlation between maternal serum IGF-1 concentrations (*P* = 0.06, *r* = 0.12), placental mRNA expression of *IGF1* (*P* = 0.05, *r* = 0.42), and birth length *z*-score (Figure 2B, C), but no association between placental expression of IGF-1R and fetal linear growth (Figure 2D). Conversely, in Guatemalan participants there was no association between maternal serum IGF-1 concentration or placental mRNA *IGF1* expression and birth length (Figure 2F, G) but the protein expression of placental IGF-1R was positively correlated (*P* = 0.0007, *r* = 0.68) with birth length *z*-score (Figure 2H). In a similar pattern in Pakistani participants, we found a nonsignificant positive association of maternal serum IGF-1 concentration (*P* = 0.19, *r* = 0.21) and a positive significant correlation of placental mRNA expression of *IGF1* (*P* < 0.01, *r* = 0.52) with birth weight *z*-score (**Supplemental Figure 2A, B**). In Guatemalan participants we found a significant correlation between placental protein expression of IGF-1R and birth weight *z*-score (*P* < 0.01, *r* = 0.68) (Supplemental Figure 2F). In Guatemalan participants, no association was found between maternal serum IGF-1 concentrations, placental mRNA expression of *IGF1*, and birth weight (Supplemental Figure 2D, E). In Pakistani participants, placental protein expression of IGF-1R was not correlated with birth weight *z*-score (Supplemental Figure 2C) and placental area was positively correlated with birth length and birth weight *z*-scores and placental mRNA expression of *IGF1* (**Supplemental Figure 3A, B, D**). In contrast, these associations were not observed in the Guatemalan cohort (Supplemental Figure 3E–H).

### Placental DNA methylation of the *IGF1* gene promoter and its association with fetal growth

**FIGURE 3:**
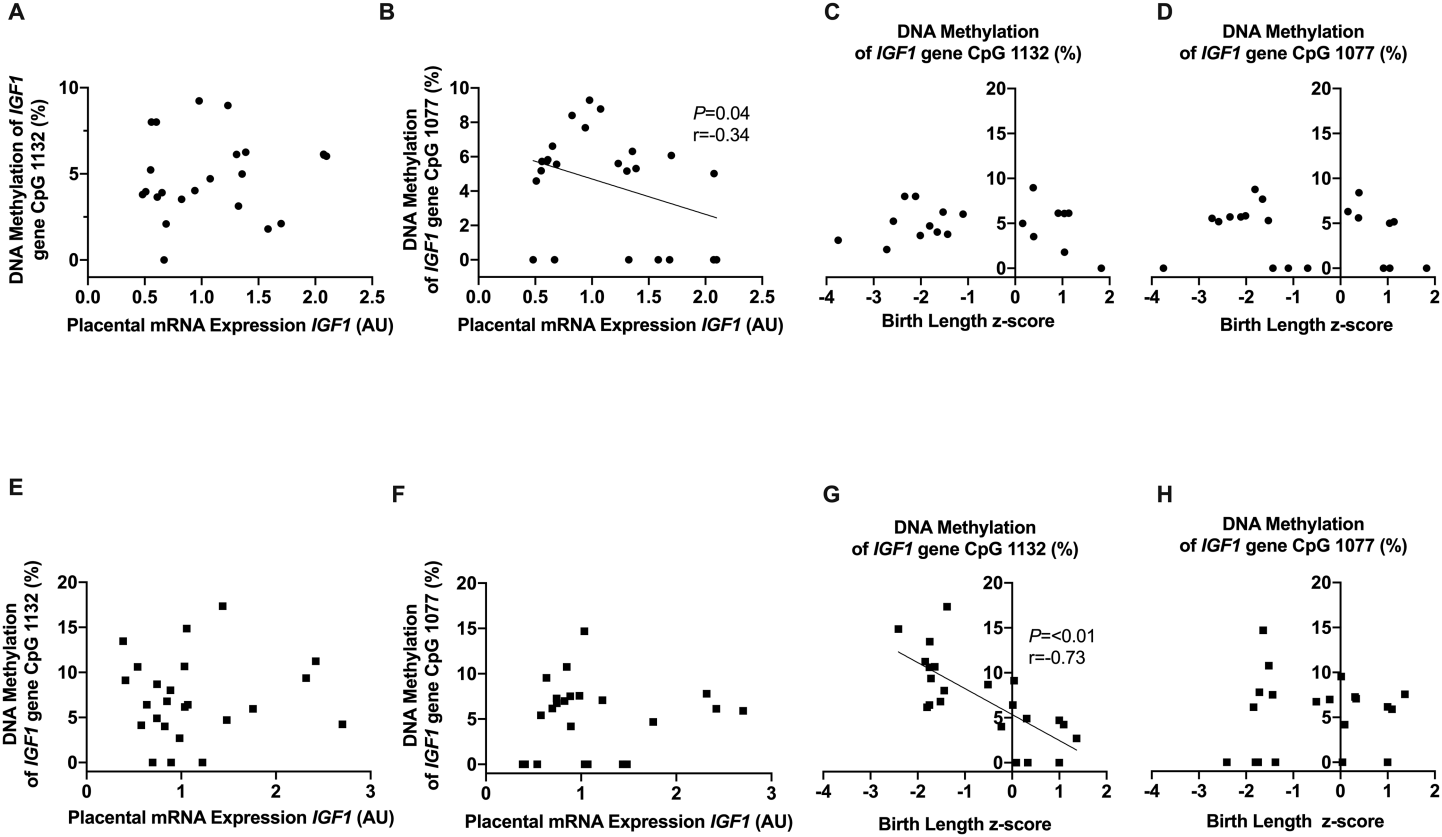
Associations between placental *IGF1* gene promoter DNA methylation, placental *IGF1* mRNA expression, and birth length *z*-score in Pakistan (A–D) and Guatemala (E–H). Pearson's correlation of placental DNA methylation of *IGF1* gene promoter at the CpG 1132 site with (A, E, *n* = 24) relative placental mRNA expression of *IGF1* and (C, *n* = 19; G, *n* = 22) birth length *z*-score. Pearson's correlation of placental DNA methylation of *IGF1* gene promoter at the CpG 1077 site with (B, F, *n* = 24) relative placental mRNA expression of *IGF1* and (D, *n* = 20; H, *n* = 22) birth length *z-*score. AU, arbitrary units; IGF-1, insulin-like growth factor-1.

Overall, no significant differences in placental DNA methylation, reported as percentage methylation, of the *IGF1* gene promoter at either CpG site were found between sites or treatment arms (CpG 1077 Guatemala: control 5.6% ± 1.0% compared with preconceptional SQLNS 4.2% ± 1.3%; Pakistan: control 4.2% ± 0.9% compared with preconceptional SQLNS 4.7% ± 0.8%; CpG 1132 Guatemala: control 7.1% ± 0.9% compared with preconceptional SQLNS 7.0% ± 1.6%; Pakistan: control 6.3% ± 1.7% compared with preconceptional SQLNS 5.0% ± 0.8%). Importantly, a significant negative correlation was observed between DNA methylation at the CpG 1077 region of the *IGF1* gene promoter and the mRNA expression of *IGF1* by the placenta for Pakistani participants only. No association existed between methylation at the CpG 1132 region and placental *IGF1* mRNA expression (**Figure 3**A, B). Birth length *z-*score was not associated with DNA methylation of the *IGF1* gene promoter at either of the CpG 1077 or CpG 1132 sites in placentas from Pakistani participants (Figure 3C, D).

In placentas from Guatemalan participants, we found no significant relation of DNA methylation of the *IGF1* gene promoter at the CpG 1077 or CpG 1132 sites with placental mRNA expression of *IGF1* (Figure 3E, F). On the other hand, we found a strong negative correlation (*P* = 0.0001) between DNA methylation of the CpG 1132 region of the *IGF1* gene promoter and birth length *z*-score (Figure 3G) and birth weight *z*-score (**Supplemental Figure 4**). Interestingly, in placentas from Guatemalan participants DNA methylation of the *IGF1* gene promoter at CpG 1077 was not associated with birth length (Figure 3H).

### Effect of preconceptional SQLNS on circulating maternal serum concentrations of IGFBP-3, IGFBP-4, and IGFBP-5 and its association with fetal growth

**FIGURE 4:**
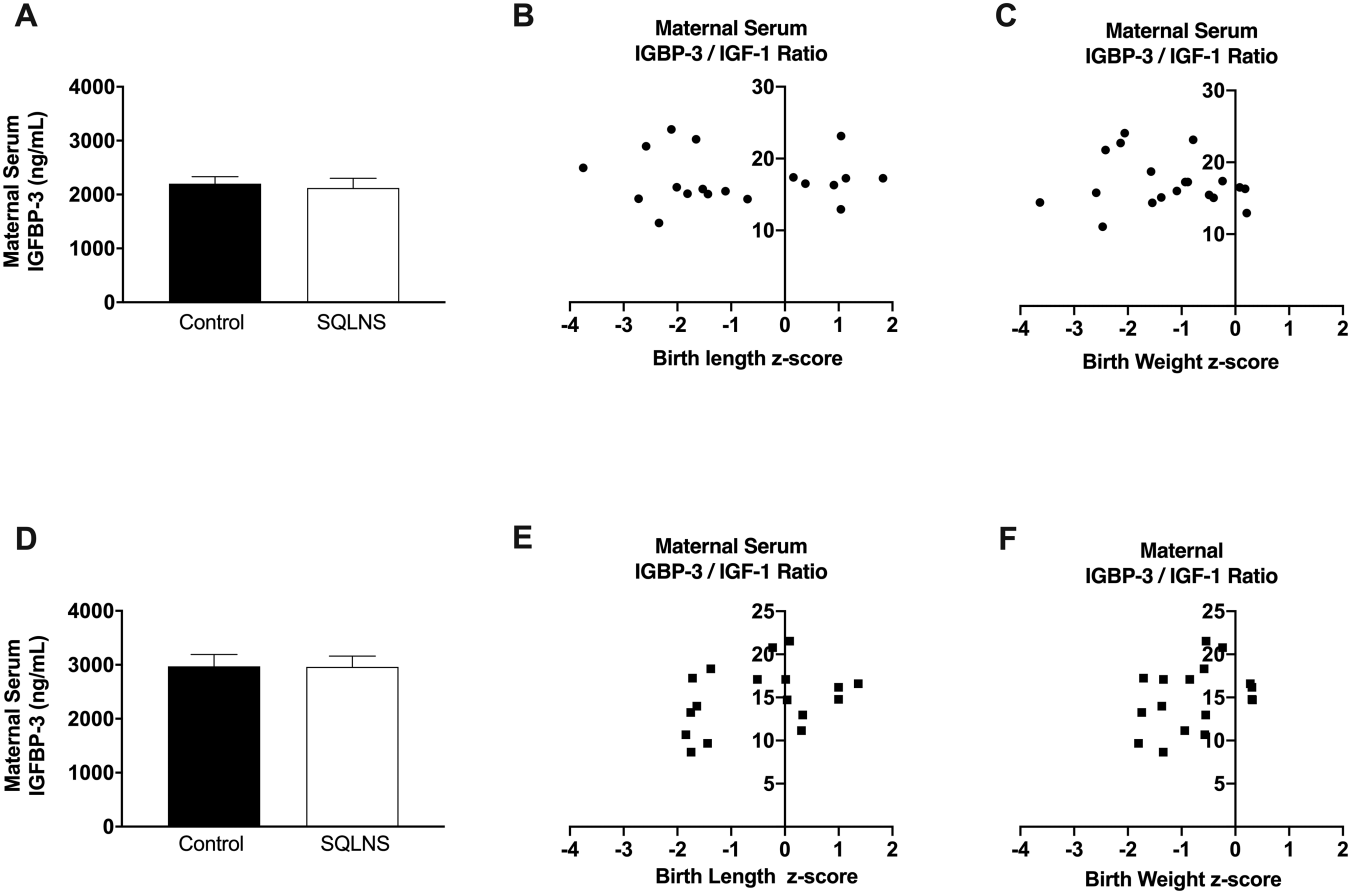
Effect of preconceptional SQLNS on maternal serum concentration of IGFBP-3 and its association with fetal growth in Pakistan (A–C) and Guatemala (D–F). The histogram shows maternal serum concentrations of IGFBP-3 by treatment arm (control, arm 3; SQLNS, arm 1) and site. Data are presented as mean ± SEM (A, *n* = 10; D, *n* = 12). Pearson's correlation between maternal concentration of IGFBP-3 and (B, *n* = 19; E; *n* = 18) birth length *z-*score and (C, *n* = 19; F, *n* = 17) birth weight *z*-score by site. IGFBP-3, insulin-like growth factor binding protein-3; IGF-1, insulin-like growth factor-1; SQLNS, small-quantity lipid-based nutrient supplementation.

**FIGURE 5:**
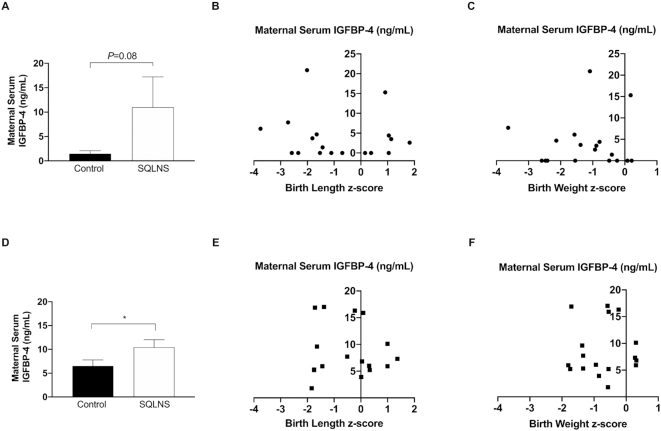
Effect of preconceptional SQLNS on maternal serum IGFBP-4 and its association with fetal growth in Pakistan (A–C) and Guatemala (D–F). The histogram shows maternal serum concentration of IGFBP-4 by treatment arm (control, arm 3; SQLNS, arm 1) and site. Data are presented as mean ± SEM (A, *n* = 11; D, *n* = 10). *Different from control, *P* < 0.05 (Student's unpaired *t* test). Pearson's correlation between maternal serum concentration of IGFBP-4 and (B, *n* = 18; E, *n* = 17) birth length *z*-score and (C, *n* = 19; F, *n* = 17) birth weight *z*-score by site. IGFBP-4, insulin-like growth factor binding protein-4; SQLNS, small-quantity lipid-based nutrient supplementation.

**FIGURE 6:**
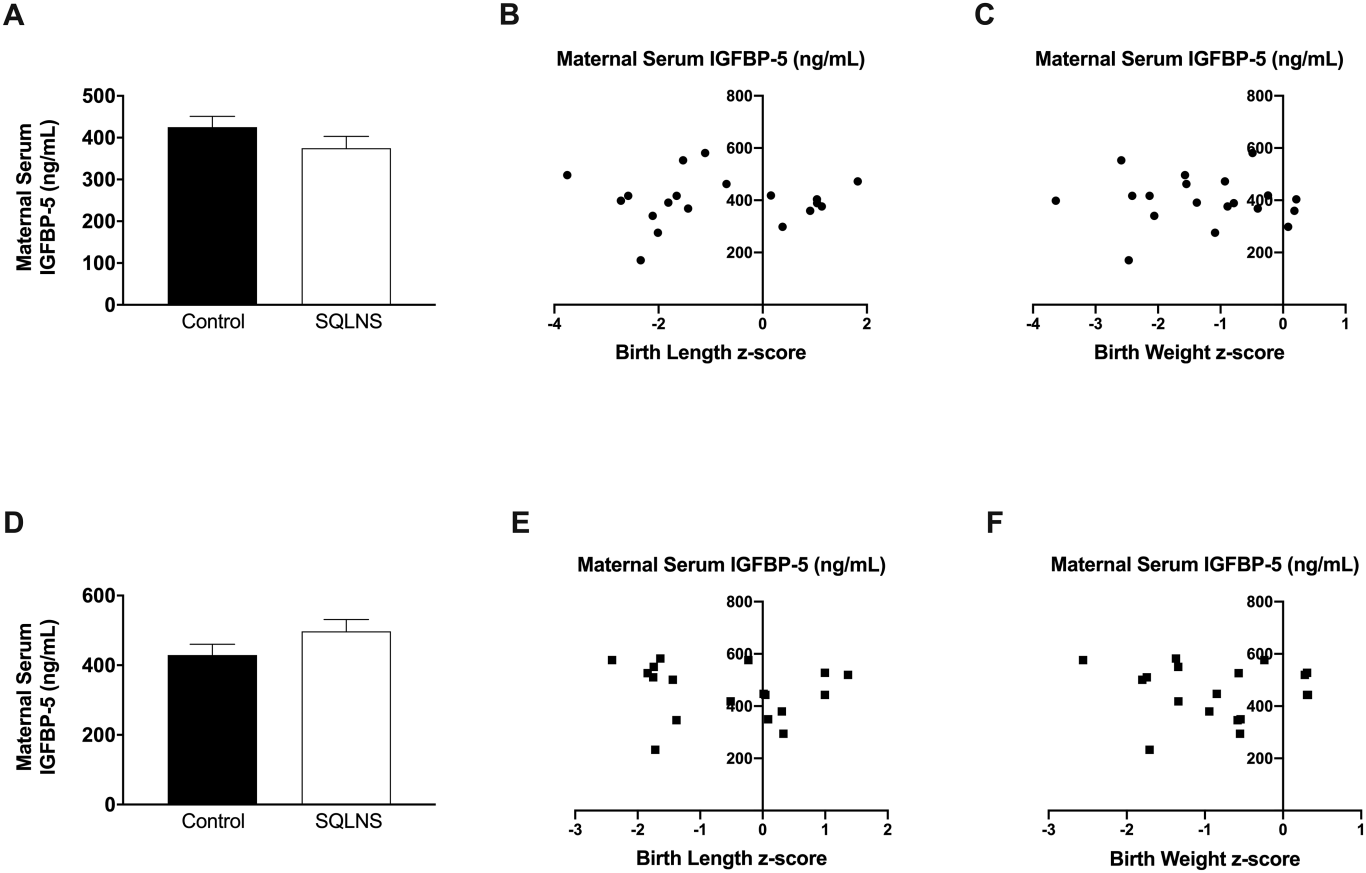
Effect of preconceptional SQLNS on maternal serum IGFBP-5 and its association with fetal growth in Pakistan (A–C) and Guatemala (D–F). The histogram shows maternal serum concentration of IGFBP-5 by treatment arm (control, arm 3; SQLNS, arm 1) and site. Data are presented as mean ± SEM (A, *n* = 11; D, *n* = 10). Pearson's correlation between maternal serum concentration of IGFBP-5 and (B, *n* = 18; E, *n* = 17) birth length *z*-score and (C, *n* = 19; F, *n* = 18) birth weight *z*-score by site. IGFBP-5, insulin-like growth factor binding protein-5; SQLNS, small-quantity lipid-based nutrient supplementation.

Preconceptional SQLNS did not affect the maternal serum concentration of IGFBP-3 in either Guatemalan or Pakistani participants, and the IGFBP-3:IGF-1 ratio was not correlated with birth length or birth weight *z*-score (**Figure 4**). In contrast, in Guatemalan participants preconceptional SQLNS significantly increased maternal serum concentration of IGFBP-4 (*P* = 0.01) and this was borderline significantly increased in Pakistani participants (*P* = 0.08). However, there was no correlation between maternal serum IGFBP-4 and birth length or birth weight *z-* scores in either cohort (**Figure 5**). IGFBP-5 was not affected by SQLNS and was not associated with fetal growth parameters in either population (**Figure 6**). In Guatemalan participants only, maternal serum IGFBP-5 was positively correlated with maternal serum zinc (*P* = 0.04, *r* = 0.40) and there was a borderline significant positive correlation between IGFBP-5 and maternal serum IGF-1 concentrations (*P* = 0.07, *r* = 0.34).

### Effect of preconceptional SQLNS on placental protein expression of PAPP-A and maternal serum zinc and its association with placental area and fetal growth

**FIGURE 7:**
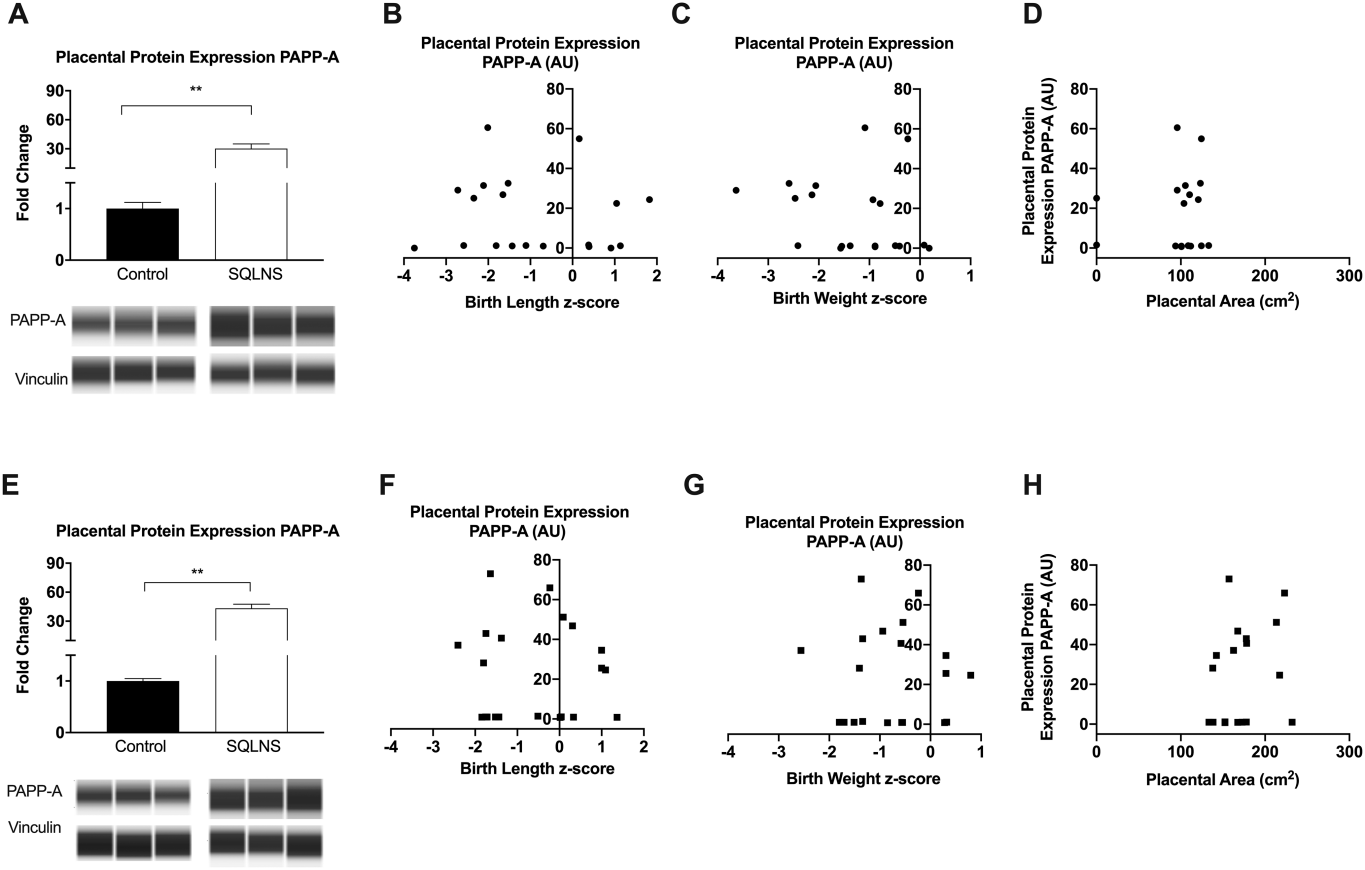
Effect of preconceptional SQLNS on placental protein expression of PAPP-A and its association with fetal growth in Pakistan (A–D) and Guatemala (E–H). The histogram shows relative placental protein expression of PAPP-A by treatment arm (control, arm 3; SQLNS, arm 1) and site (A, E). Data are presented as mean ± SEM, *n* = 12. Representative individual Protein Simple Western Blot capillaries are shown for PAPP-A and vinculin as loading control. **Different from control, *P* < 0.005 (Student's unpaired *t* test). Pearson's correlation between relative placental protein expression of PAPP-A and (B, *n* = 19; F, *n* = 22) birth length *z*-score, (C, *n* = 19; G, *n* = 22) birth weight *z*-score, and (D, *n* = 17; H, *n* = 19) placental area. AU, arbitrary units; PAPP-A, pregnancy associated plasma protein-A; SQLNS, small-quantity lipid-based nutrient supplementation.

PAPP-A modulates IGF-1 bioavailability and is released by the placenta, therefore we examined placental protein expression of PAPP-A. In both Pakistani and Guatemalan participants, preconceptional SQLNS supplementation resulted in a pronounced 50-fold increase in placental protein expression of PAPP-A (**Figure 7**A, E). Overall, a positive correlation was found between placental protein expression of PAPP-A and maternal serum concentration of IGF-1 in the preconceptional SQLNS group (*P* = 0.01, *r* = 0.47) independent of the study site. Placental expression of PAPP-A was not associated with changes in maternal serum IGFBP-3 (Guatemala: *P* = 0.33, *r* = 0.10; Pakistan: *P* = 0.22, *r* = −0.18), positively correlated with maternal serum IGFBP-4 in Guatemalan participants (*P* = 0.02, *r* = 0.46) but not in Pakistani participants (*P* = 0.10, *r* = 0.28), and positively correlated with maternal serum IGFBP-5 in Guatemalan participants (*P* = 0.05, *r* = 0.36) but not in Pakistani participants (*P* = 0.16, *r* = −0.23). We found no associations with birth length and birth weight *z*-scores or placental area in any of the study sites (Figure 7B–D, F–H).

**FIGURE 8:**
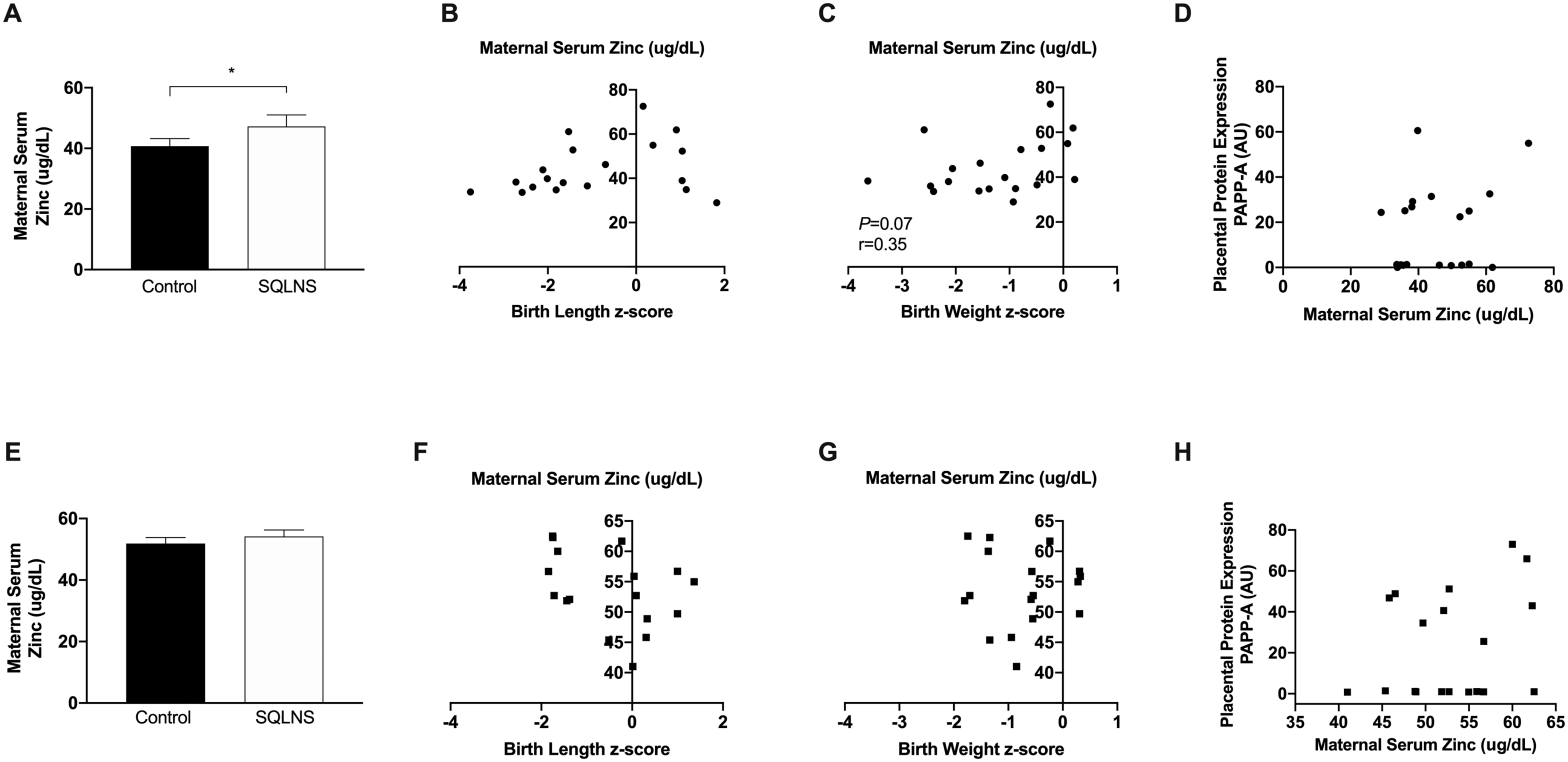
Effect of preconceptional SQLNS on maternal serum concentration of zinc and its association with fetal growth in Pakistan (A–D) and Guatemala (E–H). The histogram shows maternal serum zinc concentration by treatment arm (control, arm 3; SQLNS, arm 1) and site. Data are presented as mean ± SEM (A, *n* = 12; E, *n* = 10). *Different from control, *P < 0.05 (Student's unpaired *t* test). Pearson's correlation between maternal zinc serum concentration and (B, *n* = 19; F, *n* = 17) birth length *z*-score, (C, *n* = 19; G, *n* = 17) birth weight *z*-score, and (D, *n* = 20; H, *n* = 19) relative placental protein expression of PAPP-A. AU, arbitrary units; PAPP-A, pregnancy associated plasma protein-A; SQLNS, small-quantity lipid-based nutrient supplementation.

Because PAPP-A is a proteolytic enzyme dependent on zinc for its activity, circulating maternal serum zinc concentrations and placental zinc transporter SLC39A10 were determined. In Pakistani participants we found that preconceptional SQLNS increased maternal serum zinc concentrations (**Figure 8**) and maternal serum zinc was borderline significantly positively correlated (*P* = 0.07, *r* = 0.35) with birth weight *z*-score. In Guatemalan participants, we found no differences between treatment arms in maternal serum zinc concentrations and no associations with birth length or birth weight *z*-scores. Placental zinc transporter SLC39A10 protein expression was not affected by SQLNS in either population (**Supplemental Figure 5A, E**) and was not associated with fetal growth parameters or with placental PAPP-A protein expression in any study site (Supplemental Figure 5B–H). Only in Guatemalan participants receiving SQLNS, we found a positive association between placental zinc transporter and placental PAPP-A protein expression (SQLNS: *P* = 0.003, *r* = 0.72; Control: *P* = 0.39, *r* = 0.08).

### Effect of preconceptional SQLNS on maternal serum PAPP-A and PAPP-A2 and its association with fetal growth

Preconceptional SQLNS did not change circulating maternal concentrations of PAPP-A at 34 weeks of gestation in Pakistani (Control: 31.6 ± 4.05 mIU/mL; SQLNS: 42.9 ± 7.09 mIU/mL) or Guatemalan participants (Control: 30.5 ± 4.68 mIU/mL; SQLNS: 26.8 ± 5.90 mIU/mL). Maternal serum concentrations of PAPP-A were not associated with birth length or birth weight *z*-score, maternal serum IGF-1, zinc, or IGFBP-3, -4, and -5 in either study site (**Table 2**). In Pakistani participants preconceptional SQLNS did not change maternal concentrations of PAPP-A2 at 34 weeks of gestation (Control: 62.3 ± 9.32 ng/mL; SQLNS: 63.8 ± 17.3 ng/mL). In contrast, Guatemalan participants had significantly decreased serum concentrations of PAPP-A2 (Control: 78.7 ± 21.9 ng/mL; SQLNS: 49.1 ± 10.9 ng/mL; *P* = 0.05). In addition, in Guatemalan participants maternal serum concentration of PAPP-A2 was positively associated with birth length *z*-score (*P* = 0.01, *r* = 0.54) and with birth weight *z*-score (*P* = 0.01, *r* = 0.52). No associations were found with maternal serum IGF-1, maternal serum zinc, or IGFBP-3, -4, and -5 in either study site (Table 2).

**TABLE 2:** Associations of maternal serum PAPP-A and PAPP-A2 with fetal growth parameters and hormones, by study site^1^

	Birth length *z-*score	Birth weight *z-*score	Maternal serum IGF-1	Maternal serum zinc	Placental area	IGFBP-3	IGFBP-4	IGFBP-5
Pakistan study site								
PAPP-A	0.29	0.15	−0.02	0.32	0.31	−0.11	0.01	−0.14
PAPP-A2	0.15	−0.04	−0.08	0.18	−0.22	0.08	−0.02	0.08
Guatemala study site								
PAPP-A	−0.01	0.10	−0.22	0.14	0.55*	0.31	0.34	−0.07
PAPP-A2	0.54*	0.52*	−0.25	−0.17	−0.07	−0.24	−0.11	−0.36

1 Values are Pearson's correlation coefficients. IGFBP, insulin-like growth factor binding protein; IGF-1, insulin-like growth factor-1; PAPP, pregnancy associated plasma protein.

* *P* < 0.05.

## Discussion

In the present study, we demonstrated differential effects of a preconceptional maternal SQLNS on placental growth, function, and IGF-1 signaling in women living in low-resource countries where stunting is prevalent. In Pakistani participants, preconceptional SQLNS resulted in decreased placental AMPK activity, indicating improved cellular energy amounts and activation of placental nutrient sensing through the mTOR pathway. In Pakistani participants, maternal serum IGF-1 concentration and placental IGF-1 mRNA expression were correlated with fetal growth. In contrast, in Guatemalan participants who entered pregnancy with a greater BMI, preconceptional SQLNS did not modify placental AMPK or mTOR signaling. Placental expression and maternal serum IGF-1 concentration showed no correlation with fetal growth parameters. We propose that SQLNS effects on placental function and fetal growth in these low-income populations are dependent, in part, on maternal nutritional status defined by BMI. These distinct placental responses to SQLNS may help to explain the primary outcome in the WF trial, where preconceptional SQLNS in Pakistani participants resulted in significant increases in birth length *z*-score as compared with the control arm, with the mean effect size being substantial and similar to other reported maternal nutrition interventions. In Guatemalan participants the mean length-for-age *z-*score between the preconceptional SQLNS and control groups was not different and preconceptional SQLNS did not improve linear growth.

Fetal growth is determined by a wide variety of factors including genetic influences, maternal nutritional status and metabolism, weight gain, and placental growth and nutrient transport capacity. Many LMICs are undergoing the so-called “double burden” of malnutrition, which is characterized by growth stunting or wasting that coexists with overweight/obesity (33, 34). Because overweight and obesity are increasing rapidly in LMICs (35), this coexistence exacerbates the risk of metabolic disease later in life and the transgenerational transmission of metabolic diseases (3, 36). Maternal undernutrition is strongly associated with intrauterine growth restriction (37), downregulation of placental mTOR, and a range of placental nutrient transport systems (38–40). In the present study, half of Guatemalan participants entered pregnancy being overweight, whereas Pakistani participants were of normal to low weight. This difference in maternal nutrient reserves as measured by BMI resulted in distinct nutritional interventions according to the WF protocol. Guatemalan participants were primarily supplemented only with the SQLNS. In contrast, the majority of the Pakistani participants received both the SQLNS and a protein-energy supplement due to either low preconceptional BMI or lack of adequate weight gain during pregnancy. This difference in the nutritional intervention protocol may contribute to the distinct placental responses to the maternal supplementation observed in the 2 subcohorts.

Placental size and functional capacity are key determinants of fetal growth and can be modified by aspects of maternal nutritional status, such as BMI. Placental nutrient transporters are regulated in response to maternal nutrient supply (41). Nutrient transfer to the fetus is dependent on the transport surface area and placental size is a surrogate measure of surface area (11). Placental weight as a measure of placental size is correlated closely with birth weight (42). In our study placental weight was not measured; however, Guatemalan participants had greater placental area than Pakistani participants. This is in agreement with studies showing that higher maternal BMI is associated with increased placental size (43, 44). Interestingly, a significant increase in placental area was found in Pakistani participants who received SQLNS as compared with control participants, and when SQLNS and control groups were pooled, a positive correlation between placental area and birth length and birth weight *z*-scores was found. Prior studies have shown that placental weight and the ratio of placental weight to birth weight are predictive of maternal disease, perinatal morbidity and mortality, as well as childhood growth and development (45, 46). Thus, placental growth and function are key mediators of adequate intrauterine fetal growth and lifelong health. Our study demonstrates the interaction between maternal nutrient status, nutrient supplementation, and placental size in women with low nutrient reserves who are supplemented during pregnancy.

### Nutrient-sensing pathway intrinsic to the placenta

Placental nutrient transporters are regulated in response to maternal nutrient supply (47). Nutrient-sensing pathways in the placenta include mTOR signaling, which has been shown to regulate placental nutrient transporter expression and activity (13, 48). Previous studies in animal models and human pregnancies have demonstrated that mTOR regulates placental nutrient transporters in response to a wide variety of signals, including growth factors such as IGF-1. Notably, placental mTOR activity is decreased in placentas of intrauterine-growth-restricted human infants (49). Inhibition of mTOR markedly decreases the activity of key placental amino acid transporters in cultured primary human trophoblast cells (13, 48). Therefore, dysregulation of placental mTOR plays an important role in abnormal fetal growth by modulating the delivery of nutrients to the fetus (48).

Two distinct patterns of placental mTOR activity were observed in women who received preconceptional SQLNS. In underweight Pakistani participants, preconceptional SQLNS combined with protein-energy supplementation stimulated placental mTOR signaling. In Guatemalan participants with greater caloric reserves and high rates of maternal stunting, preconceptional SQLNS did not affect placental mTOR signaling. We propose that these distinct responses are due, in part, to differences in maternal nutritional status, and possibly modulated by the differences in the interventional protocol in these 2 sites. Our data suggest that, in Pakistani participants, preconceptional SQLNS together with a protein-energy supplement activated placental mTOR and IGF-1 signaling, likely increasing placental growth and nutrient transfer to improve fetal growth. Given that mTOR is particularly responsive to amino acid availability, it cannot be excluded that the additional protein-energy supplement provided to the Pakistani participants contributed to placental mTOR activation and increased fetal growth. In Guatemalan participants, SQLNS did not activate placental mTOR or IGF-1 signaling and did not promote fetal growth.

### IGF-1 axis and regulation of fetal growth

Maternal hormones such as insulin, leptin, adiponectin, and IGF-1 are factors that regulate placental transport through mTOR signaling. IGF-1 is known to modulate placental transport of nutrients and provides a link between maternal nutritional status and placental nutrient transport (50). Contrary to our hypothesis, maternal preconceptional SQLNS did not affect the circulating maternal serum concentration of IGF-1 nor DNA methylation of the placental *IGF1* gene promoter in either study site. We observed that maternal serum IGF-1 and placental mRNA expression of *IGF1* were positively correlated with birth size parameters in Pakistani participants. In contrast, in Guatemalan participants we found a positive correlation of placental protein expression of IGF-1R with fetal growth but not with maternal serum IGF-1 or placental expression. These data suggest important differences in the regulation of intrauterine growth in Guatemalan and Pakistani women, a concept supported by the distinct relations between placental *IGF1* gene promoter DNA methylation and fetal growth in the 2 cohorts. Specifically, in Guatemala, but not in Pakistan, we found a striking negative relation between placental DNA methylation of the *IGF1* gene promoter and fetal growth. Previous studies have shown that, in small-for-gestational-age infants, placental *IGF1* mRNA expression was decreased and corresponded to hypermethylation of the *IGF1* gene promoter, indicating that epigenetic modification of the placental *IGF1* gene may regulate fetal growth (24). In the placentas of Guatemalan participants, DNA methylation of the *IGF1* gene promoter at the 2 studied CpG sites was not associated with changes in placental *IGF1* mRNA expression, suggesting the impact of this change in methylation on fetal growth may not be due to direct changes in IGF-1 concentration but may operate through an alternative, as yet unidentified, pathway.

IGF activity is regulated through interaction with IGFBPs and proteases such as PAPP-A and PAPP-A2 that cleave IGFBPs, providing an additional layer of complexity for modulating the bioavailability of IGFs. IGFBP-4 modulates IGF bioavailability and is a primary substrate for PAPP-A. Cleavage of the binding protein causes the release of IGF-1, allowing it to interact with receptors. In human pregnancy, PAPP-A is synthesized by the syncytiotrophoblast and circulating concentrations of PAPP-A increase across gestation as a reflection of increasing placental size (51). Reduced maternal concentrations of PAPP-A have been associated with pre-eclampsia and intrauterine growth retardation (52). Higher concentrations in the first trimester of pregnancy have been associated with large-for-gestational-age infants (20). Recently, positive associations of umbilical cord blood total IGFBP-4 and IGFBP-5 with birth length and birth weight *z*-scores have been demonstrated (53). We found an increase in maternal serum IGFBP-4 and in placental expression of PAPP-A with preconceptional SQLNS in both populations. In contrast, we did not find associations of maternal serum IGFBP-3, IGFBP-4, IGFBP-5, and PAPP-A with fetal growth parameters in either studied population. PAPP-A and PAPP-A2 in umbilical cord blood have been negatively associated with birth weight and birth length *z*-scores (53). In our study populations, maternal serum PAPP-A was not associated with fetal growth parameters. Only Guatemalan participants were found to have a positive association between PAPP-A2 and birth length and birth weight *z*-scores.

Zinc is an essential micronutrient during pregnancy and throughout life. Maternal zinc deficiency impairs fetal embryogenesis, growth, and placental development. However, the exact mechanisms are unclear. Our data suggest that in Pakistani participants serum zinc concentrations were improved with preconceptional SQLNS, which may contribute to the observed increase in birth length *z-*score reported in the larger WF trial (54). Zinc is an important cofactor for PAPP-A activity. Improved maternal zinc concentration in Pakistani participants receiving SQLNS may in part explain the response to IGF-1 signaling. The interaction of zinc with IGFBPs to regulate IGF signaling has been described in myoblasts by lowering the affinity of soluble IGFBP-5 for IGF-1 and thus increasing the availability of IGF-1 (55). In our study, only Guatemalan participants had positive associations of maternal serum IGFBP-5 with maternal serum IGF-1 and zinc. Whether increased zinc availability impinges on signaling pathways known to regulate placental function, such as mTOR and IGF-1 signaling, remains to be established.

Zinc influx (Slc39/ZIP, members 1–14) proteins transport zinc into the cell when zinc in the cytosol is low or depleted. Zinc transporters have been implicated in the activation of key cell signaling molecules associated with insulin signaling. In our study, placental zinc transporter SLC39A10 protein expression was not affected by SQLNS in either study site but was positively associated with placental PAPP-A in Guatemalan women receiving SQLNS. The role of the placenta in regulating micronutrient transport in response to maternal nutrient status is still under investigation. It is known that the placenta upregulates gene expression of zinc uptake transporters in order to meet fetal demands under low maternal zinc concentrations (56).

Functions of PAPP-A unrelated to IGF signaling are also possible and may rely on the cleavage of substrates other than IGFBPs, or a function independent of its proteolytic activity associated with early development (57). Although PAPP-A has been associated with fat deposition and body composition (58, 59), we did not observe an association between placental PAPP-A and weight gain at 34 weeks of gestation in this subset of participants at either study site (Guatemala: *P* = 0.45, *r* = 0.02; Pakistan: *P* = 0.45, *r* = 0.02).

### Strengths and limitations

A strength of this study is the opportunity to evaluate the effect of a preconceptional nutritional intervention on placental function and fetal growth in 2 low-resource populations with different maternal anthropometric measures related to nutritional status. We acknowledge that the small sample size limits our statistical power in some of the observed associations and does not necessarily represent the cohorts as a whole. Sex differences in fetal growth trajectories and sex-specific risk factors have been recognized (60) but we were unable to specifically study the effect of fetal sex on our outcomes; a balance of female and male newborns was included in the analysis to ensure fetal sex dimorphism was not contributing to the current findings. We also acknowledge the genetic background of the 2 populations might have influenced the response. The WF trial will more broadly evaluate the epigenome of mother, fetus, and offspring in future studies.

### Conclusion

**FIGURE 9:**
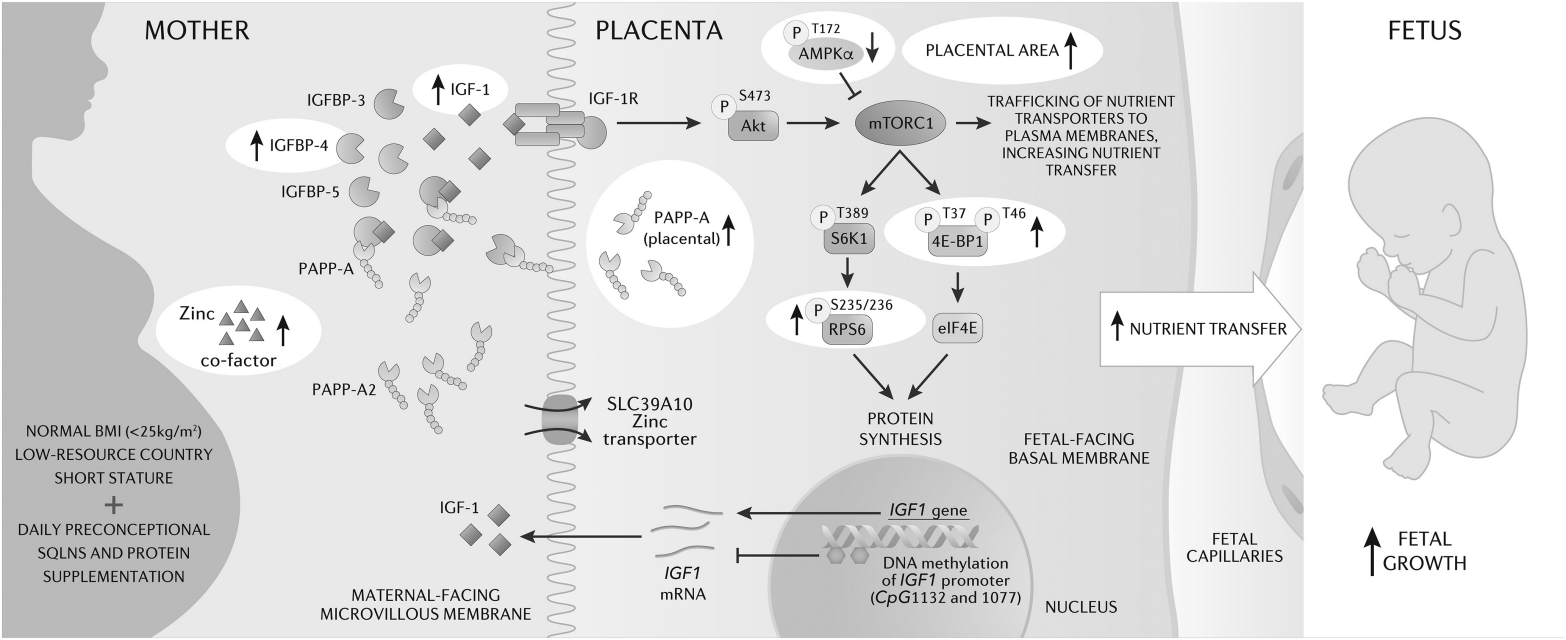
Integrative model of examined variables in the maternal circulation and in the placenta. Representation of examined variables in the maternal circulation (left) associated with the regulation of the IGF-1 pathway: IGF-1; IGFBP-3, IGFBP-4, and IGFBP-5; PAPP-A and PAPP-A2; and zinc. Description of examined variables in the placenta (61) associated with the IGF-1/mechanistic target of rapamycin pathway and fetal growth: mRNA expression of *IGF1*; IGF-1R; AMPKA; rpS6; 4E-BP1; DNA methylation of *IGF1* promoter at the CpG 1132 and 1077 sites; and SLC39A10. Study main findings are highlighted by circles. AMPKA, AMP-activated protein kinase α; Akt, protein kinase B; eIF4E, eukaryotic translation initiation factor 4E; IGFBP, insulin-like growth factor binding protein; IGF-1, insulin-like growth factor-1; IGF-1R, insulin-like growth factor 1 receptor; mTORC1, mechanistic target of rapamycin complex 1; PAPP, pregnancy-associated plasma protein; rpS6, ribosomal protein S6; SLC39A10, zinc influx SLC39A transporter; S6K1, ribosomal protein S6 kinase β-1; 4E-BP1, eukaryotic translation initiation factor binding protein-1.

In conclusion, this study provided evidence of differential effects of preconceptional SQLNS on placental function. In addition, we investigated whether IGF-1 and mTOR signaling were associated with fetal growth in low-resource populations with high rates of stunting. Preconceptional SQLNS enhanced with protein-energy supplementation in Pakistani participants who entered pregnancy with lower nutritional reserves improved placental mTOR activity and IGF-1 signaling and improved fetal growth. In contrast, preconceptional SQLNS had no effect on placental mTOR or IGF-1 signaling in Guatemalan participants with greater caloric reserves and did not improve intrauterine growth. This study suggests that in undernourished women, providing supplemental nutrition activates the placenta and improves fetal growth. Guatemalan participants did not demonstrate a placental response to the SQLNS and the classically accepted fetal growth regulation by the IGF-1 system could not be demonstrated (**Figure 9**). This study reinforces the importance of maternal supplementation on the regulation of placental and maternal IGF-1 signaling in regulating fetal growth and emphasizes that the nature of the supplement and the nutritional status of the mother must be considered. These initial studies of placental responses to maternal supplementation indicate the need for more comprehensive studies to determine the role of the placenta in determining supplement effectiveness, and if confirmed may provide insight into optimal preventive management of stunting in low-resource countries.

## Supplementary Material

nxaa354_Supplemental_File

## Notes

Supported by Bill & Melinda Gates Foundation grant OPP1055867 (to NFK) and Eunice Kennedy Shriver National Institute of Child Health and Human Development and Office of Dietary Supplements grants NIH U10 HD 076474 (to NFK) and HD 068370 (to TLP). The Bill & Melinda Gates Foundation had no role in study design, data collection, data analysis, data interpretation, or the writing of this article. The content is solely the responsibility of the authors and does not necessarily represent the official view of the NIH.

Author disclosures: The authors report no conflicts of interest.

Supplemental Table 1 and Supplemental Figures 1–5 are available from the “Supplementary data” link in the online posting of the article and from the same link in the online table of contents at https://academic.oup.com/jn/.

Abbreviations used: AMPKA, AMP-activated protein kinase α; IGFBP, insulin-like growth factor binding protein; IGF-1, insulin-like growth factor-1; IGF-1R, insulin-like growth factor receptor; LBW, low birth weight; LMIC, low- and middle-income country; mTOR, mechanistic target of rapamycin; PAPP, pregnancy associated plasma protein; rpS6, ribosomal protein S6; SLC39A10, zinc influx SLC39A transporter; SQLNS, small-quantity lipid-based nutrient supplementation; WF, Women First Preconception Maternal Nutrition Trial; 4E-BP1, eukaryotic translation initiation factor binding protein-1.
