# Supplementary material for: Preconceptional Lipid-Based Nutrient Supplementation in 2 Low-Resource Countries Results in Distinctly Different IGF-1/mTOR Placental Responses
Source: J Nutr. 2020 Dec 31;151(3):556–69. doi: 10.1093/jn/nxaa354 (PMC7948206; doi:10.1093/jn/nxaa354)

## Slide 1
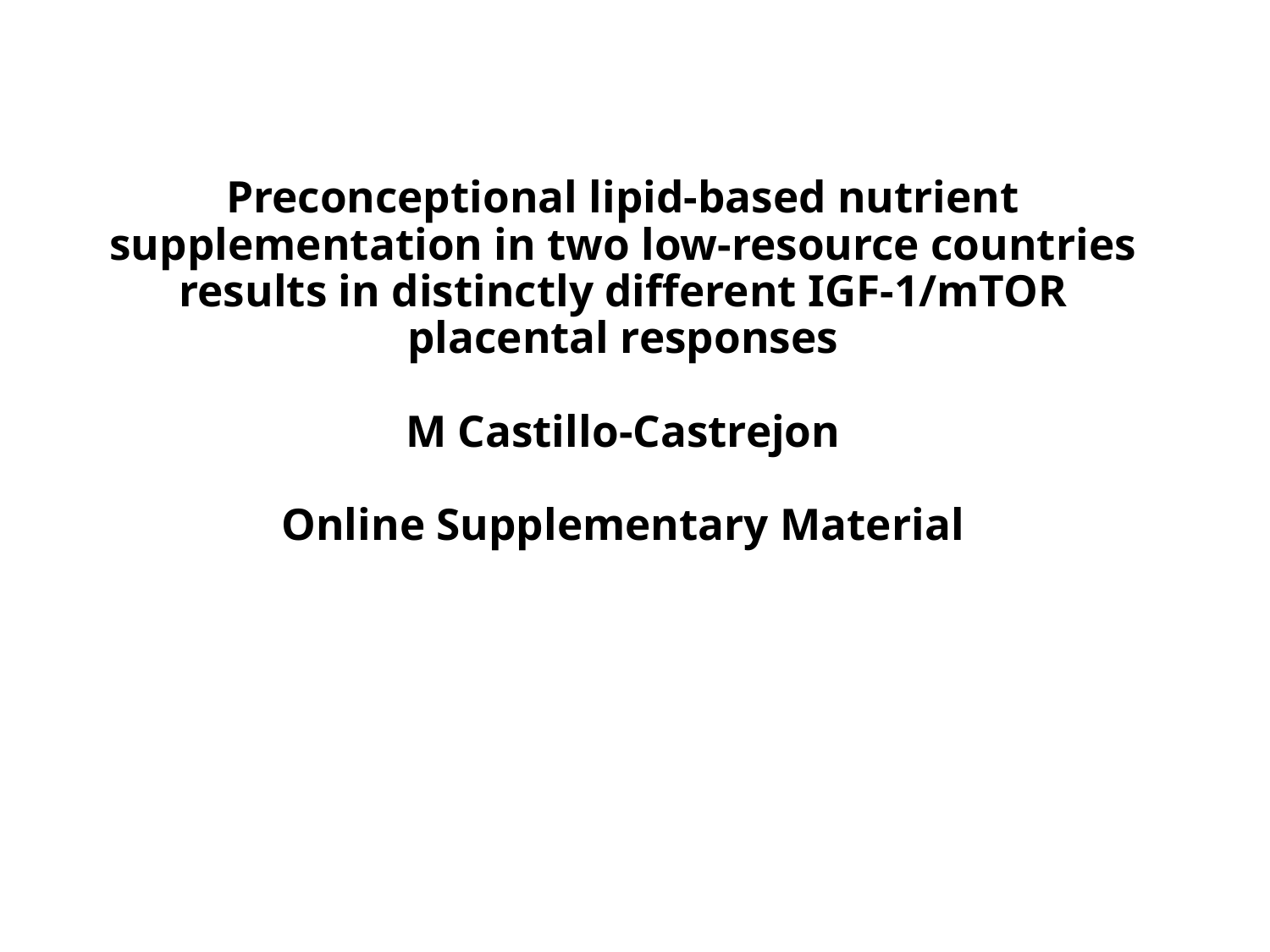

# Preconceptional lipid-based nutrient supplementation in two low-resource countries results in distinctly different IGF-1/mTOR placental responses M Castillo-Castrejon Online Supplementary Material

## Slide 2
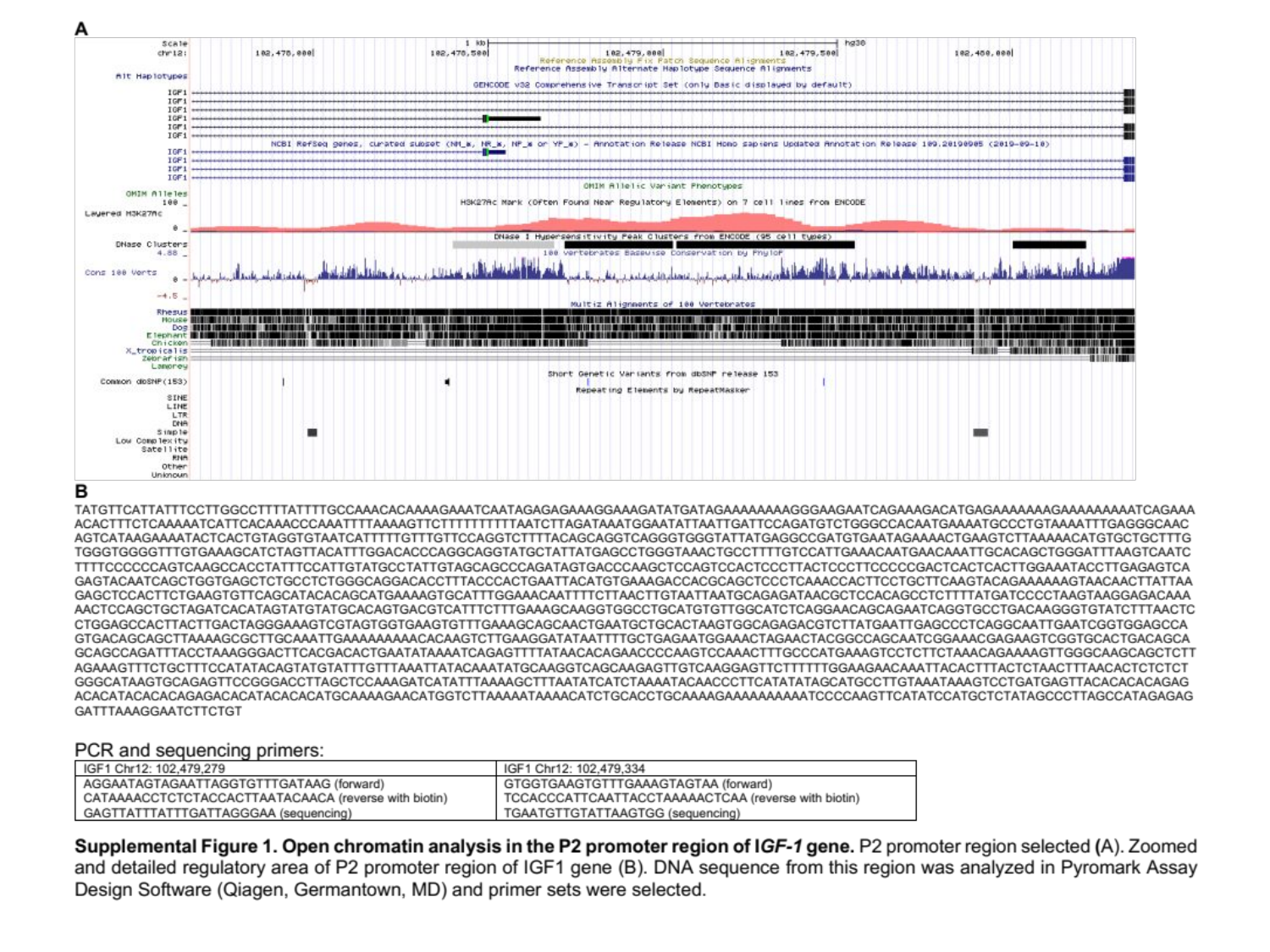

## Slide 3
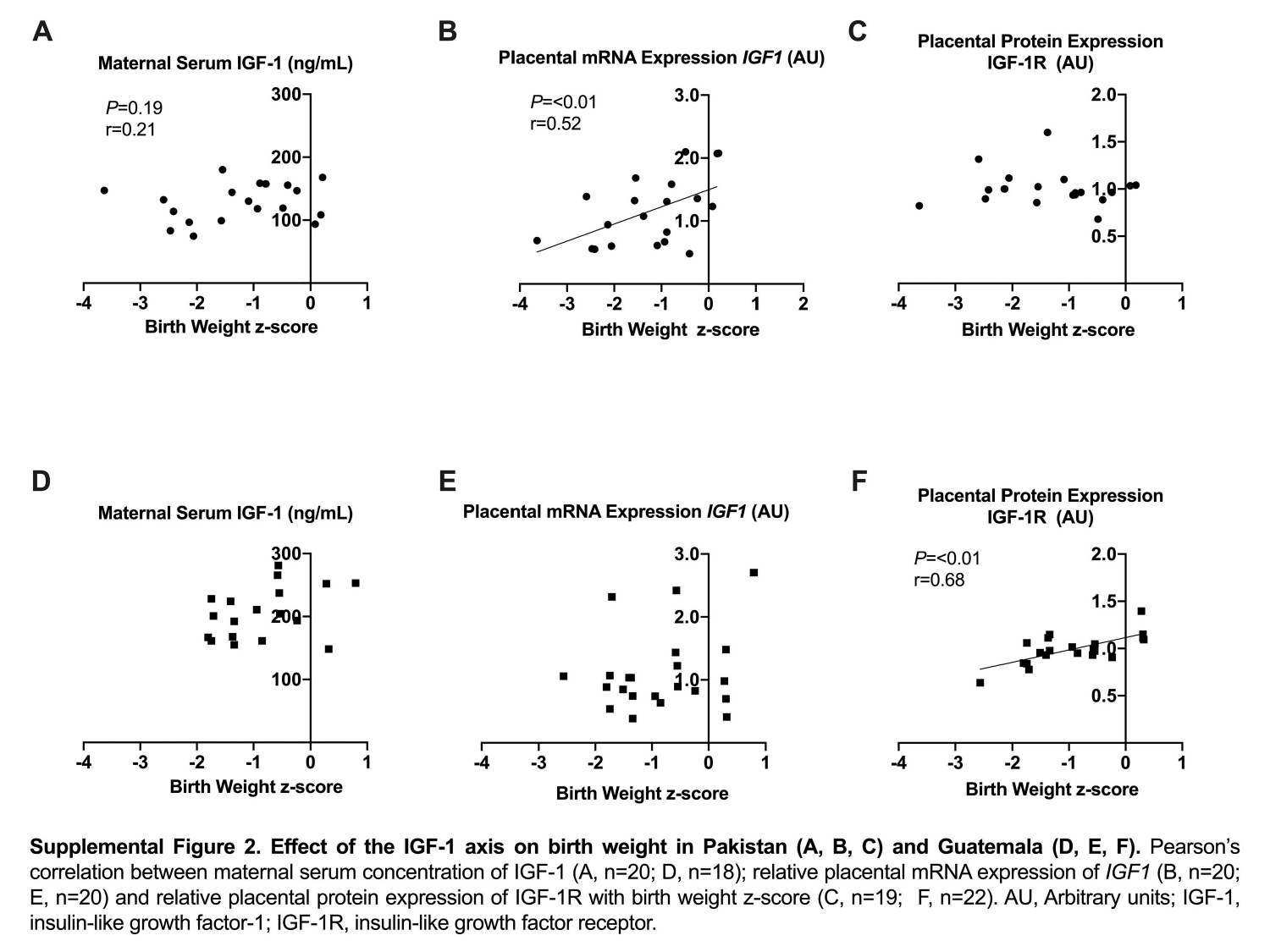

## Slide 4
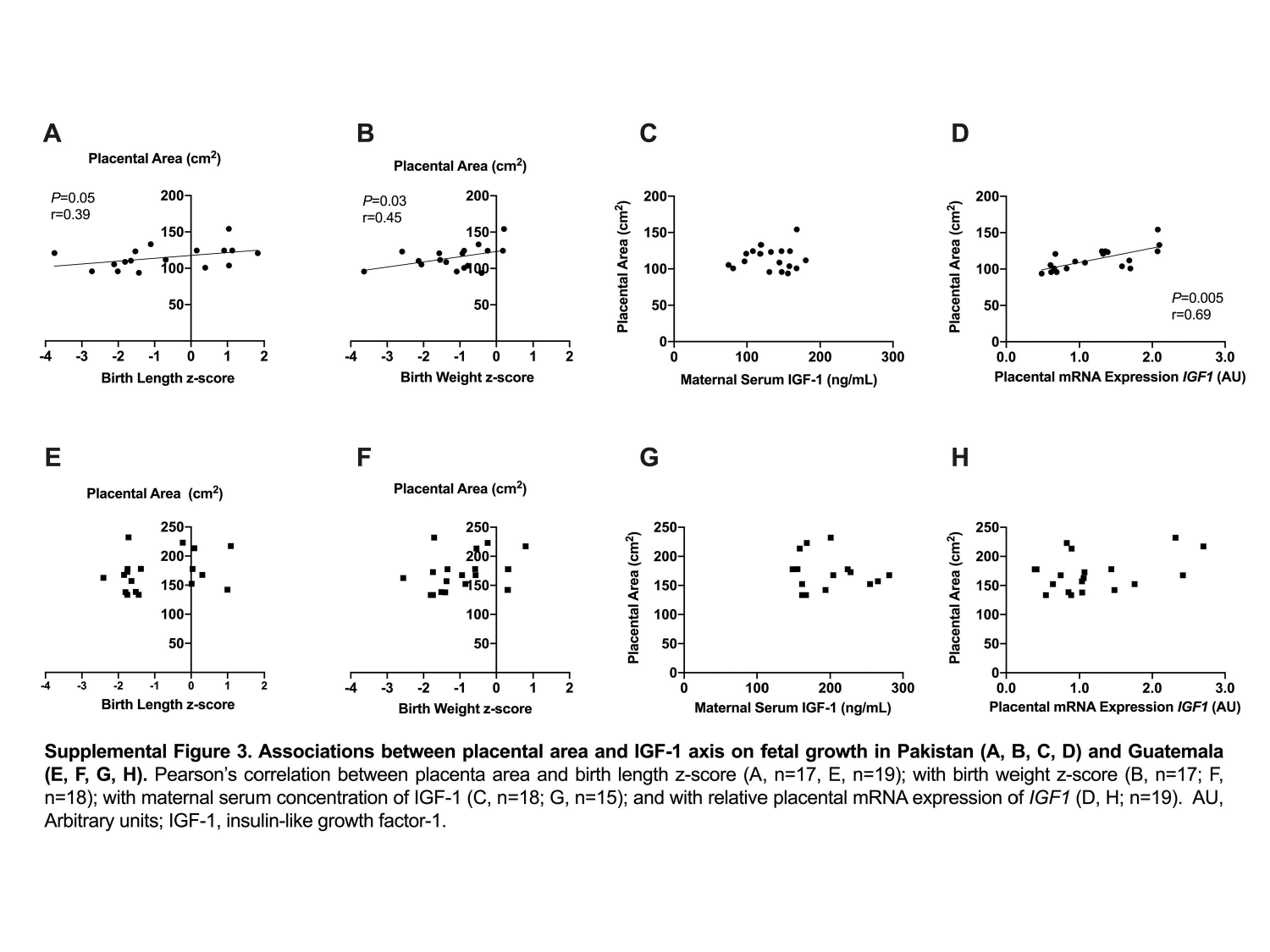

## Slide 5
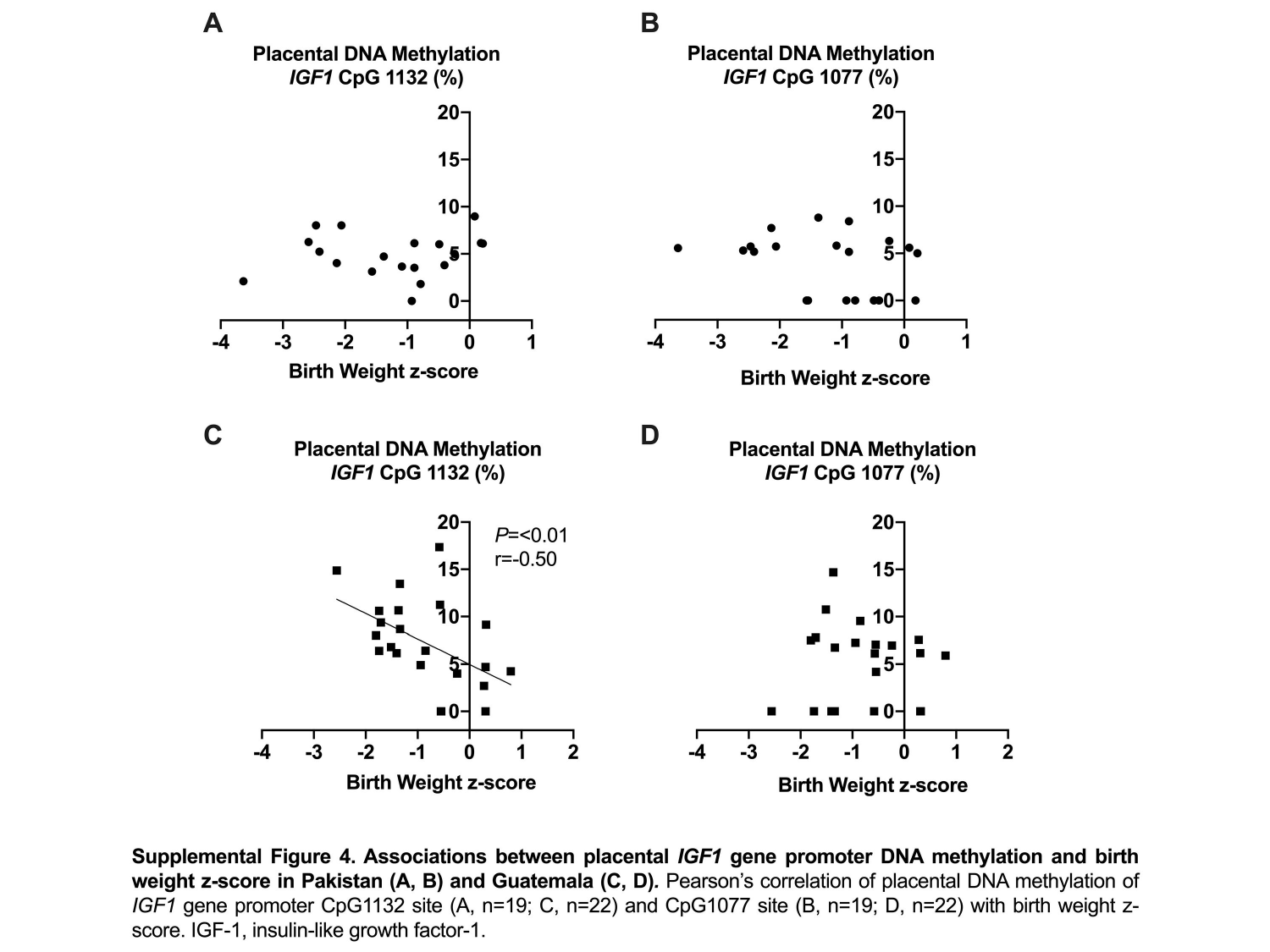

## Slide 6
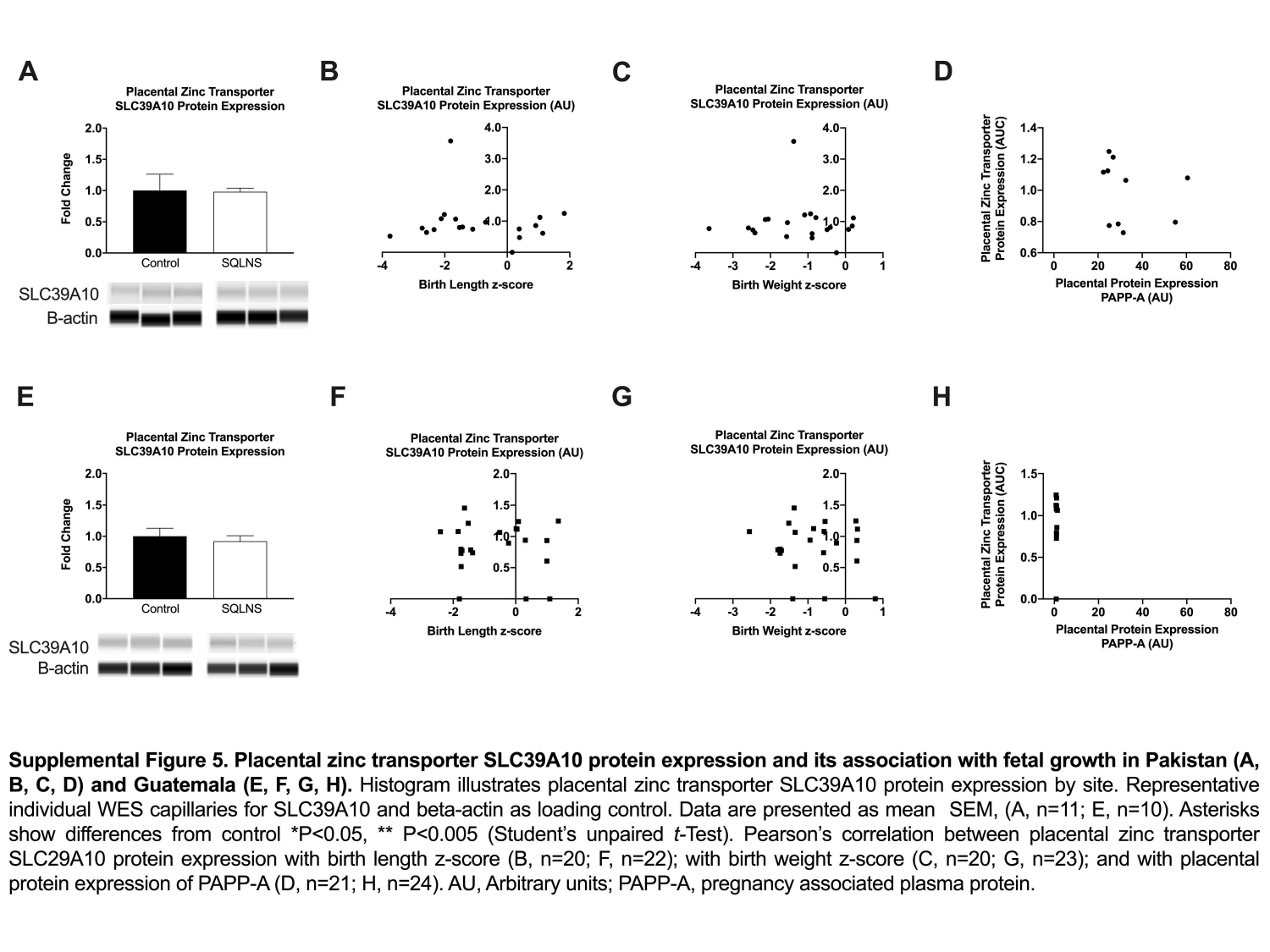

Supplement: nxaa354_Supplemental_File [file nxaa354_supplemental_file.zip › Supplemental_material_(Figures_1-5) dec2020.pptx]
